# Catamenial pneumothorax revealing diaphragmatic endometriosis: a case report and revue of literature

**DOI:** 10.11604/pamj.2017.27.112.8007

**Published:** 2017-06-14

**Authors:** Sana Aissa, Wafa Benzarti, Faouzi Alimi, Imen Gargouri, Halima Ben Salem, Amène Aissa, Khadija Fathallah, Atef Ben Abdelkade, Rafika Alouini, Abdelhamid Garrouche, Abdelaziz Hayoun, Ahmed Abdelghani, Mohamed Benzarti

**Affiliations:** 1 Pneumology Department, Farhat Hached Hospital, Sousse, Tunisia; 2 Cardiac and Thoracic Surgery Department, Sahloul Hospital, Sousse, Tunisia; 3 Radiology Department, Ibn Jazzar Hospital, Kairouan, Tunisia; 4 Gynecology and Obstetrics Department, Farhat Hached Hospital, Sousse, Tunisia; 5 Anatomic Pathology Department, Farhat Hached Hospital, Sousse, Tunisia

**Keywords:** Catamenial pneumothorax, endometriosis, surgical treatment

## Abstract

Catamenial pneumothorax (CP) is a rare entity of spontaneous, recurring pneumothorax in women. We aim to discuss the etiology, clinical course, and surgical treatment of a 42-year-old woman with CP. This patient had a right-sided spontaneous pneumothoraces occurred one week after menses. She had under-gone video-assisted thoracoscopic surgery (VATS) because of a persistent air leak under chest tube. VATS revealed multiple diaphragmatic fenestrations with an upper right nodule. Defects were removed and a large part of the diaphragm was resected. Pleural abrasion was then performed over the diaphragm. Diaphragmatic endometriosis was confirmed by microscopic examination. Medical treatment with GnRH agonists was prescribed, and after recovery, the patient has been symptoms free for 20 months.

## Introduction

Catamenial pneumothorax (CP) is a recurrent spontaneous pneumothorax (SP) that occurs in women within 72 hours of the onset of menstruation [1, 2]. CP is associated with pelvic endometriosis in 30-50% of cases. Video-assisted thoracoscopic management preferably performed during menstruation allows full inspection of the diaphragm seeking for defects and explore the entire pleural cavity looking for ectopic lesions at the level of parietal pleural and lung endometrial nodules and bullae. [3].

## Patient and observation

**Figure 1:**
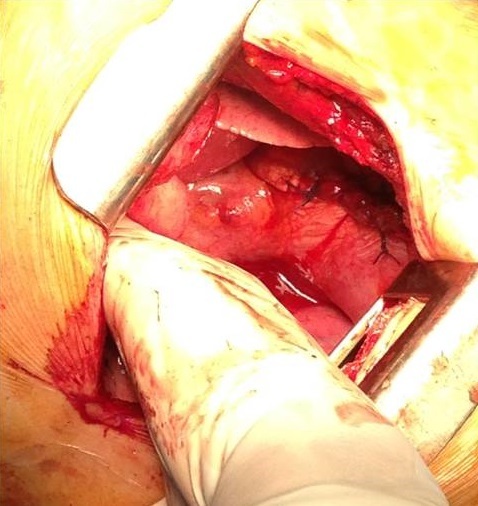
Fenestrated patch of diaphragm taking diaphragmatic slits

**Figure 2:**
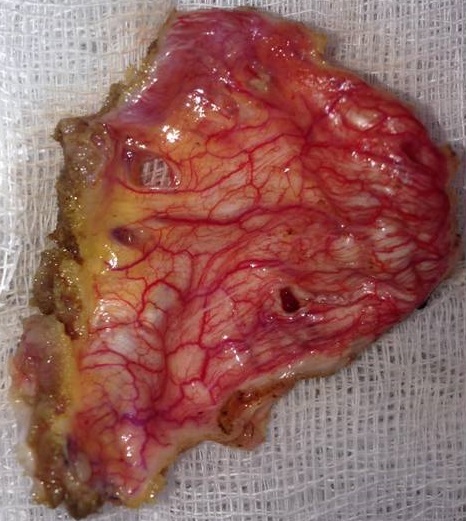
Anatomical view of the diaphragm after resection of pathological patch and repair of the diaphragm by separate points

In November 2013, a healthy 42-year-old non smoker woman with a history of secondary infertility unexplored, presented in the emergency department with a spontaneous right sided chest pain associated with dyspnea occuring one week after menses. The X-ray chest revealed a right pneumothorax. The initial treatment consisted on the insertion of an intercostal chest drain. Because of a persistent air leak under chest tube after 10 days, Video-Assisted Thoracoscopic Surgery (VATS) was performed and revealed multiple diaphragmatic fenestrations with an upper right nodule. A large part of the diaphragm was resected, removing the defects associated with a wedge resection of the nodule. Pleural abrasion was then performed over the diaphragm (Figure 1, Figure 2).

**Figure 3:**
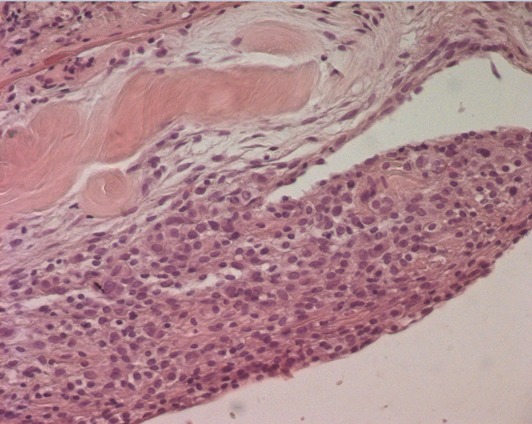
HE stain x 400: endometrial tissu dissociating diaphragmatic muscular fibers

**Figure 4:**
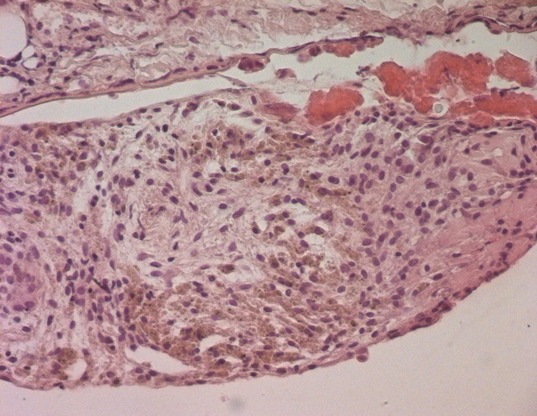
HE stain x 400: siderophages (brown spots) within endometriosic focus

**Figure 5:**
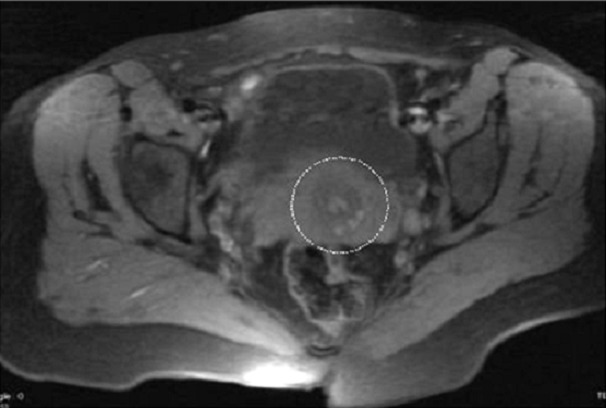
Pelvic MRI: axial sequence (T1 FATSAT) evidenced multiple round lesions in hypersignal T1 facing the cervical vaginal area

**Figure 6:**
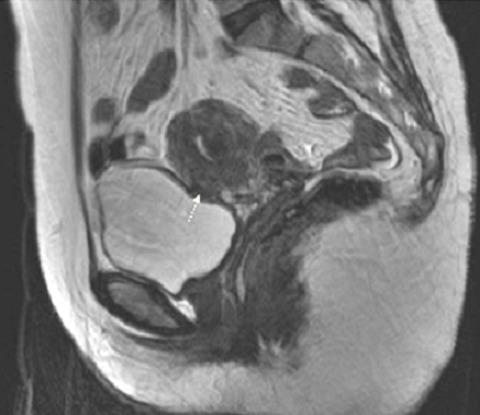
Pelvic MRI: sagittal sequence (T2 FSE) evidenced an oblong nodular lesion in hyposignal T2 in front of the vesicouterine cul de sac

The patient's recovery was uneventful and she was discharged home few days later. Samples of the pulmonary and the diaphragmatic nodules were performed. Microscopically, the latest one showed a small endometrial focus including siderophages dues to former hemorrhages (Figure 3, Figure 4). The pulmonary nodule corresponds to a fibrous scar tissue. Two months after surgery, the patient was followed and was not symptomatic at the time of her menstruations. Pelvic and chest MRI were performed as part of the assessment of the lesions of the disease. Chest MRI revealed no other diaphragmatic foci. Pelvic MRI revealed in both axial and sagittal sequences suggestive lesions of pelvic endomtriosis (Figure 5, Figure 6). Medical treatment with GnRH agonists was prescribed. After recovery, the patient has been symptoms free for 20 months.

## Discussion

Thoracic endometriosis is rare. Its prevalence has been underestimated in the literature until the last decade and several studies have investigated the diagnostic entity [4]. Endometriosis affects 5% to 15% of women in reproductive age [1]. Several hypotheses have been raised to explain this pathology [5]. A review of autopsy data showed that pulmonary endometriosis is usually bilateral while pleural and diaphragmatic endometriosis lesions were always on the right side. These led to a new hypothesis which is the most supported, that parenchyma lesions come from the lymphatic or vascular embolization while pleural and diaphragmatic injuries are the result of transdiaphragmatic or trans peritoneal migration of endometrial tissue [6]. Thoracic endometriosis is associated with a variety of symptoms but none of which is pathognomonic. In 90 % of cases, symptoms appear 24-48 hours after the onset of menstruation, and may be delayed up to 96 hours [7, 8]. Concordance between respiratory symptoms during menstruation is highly suggestive but not always found. The four main clinical entities are the catamenial pneumothorax, hemothorax isolated or associated with a pneumothorax, hemoptysis and the solitary pulmonary nodule [3]. The catamenial pneumothorax is the most common form of presentation and can occur simply in form of chest pain. Symptoms are non-specific, they are generally well tolerated, but severe presentations have been described [9]. Although the temporal relationship with menstruation is characteristic, inter menstrual episodes have been reported [10]. Pneumothorax is usually small on the chest X-Rays [7]. Chest X-Rays and CT did not identify endometrial origin of pneumothorax. Chest CT has a limited value for the diagnosis of CP but it can help providing differential diagnosis and reveals associated pneumoperitoneum or diaphragmatic endometrial implants [11]. Helical CT with reconstruction and virtual endoscopy can identify strictly endobronchial endometriosis lesions [12]. Chest MRI is superior to CT in displaying diaphragmatic endometriosis lesions, thanks to its higher contrast resolution and better characterization of haemorrhagic lesions [13]. Chest MRI can also detect small pleural endometriosis characterized by the presence of small cystic hyperintense lesions on T1-weighted images of the visceral or parietal pleura [14, 15].

In our case, the chest MRI was performed after the surgery in order to detect residual diaphragmatic lesions that may lead to recurrent pneumothorax and no abnormalities were found. Previous studies reported concurrent pelvic endometriosis in approximately 50% to 80% of cases as in our case [7]. The investigation should search pelvic pain and infertility history and so, a pelvic exam is recommended including pelvic imaging. Thoracic endometriosis requires a multidisciplinary approach that combines a hormonal medical treatment and surgery [1, 11]. VATS is the gold standard for the diagnosis of thoracic endometriosis. It allows histological diagnosis of endometriosis lesions, resection of the diaphragm if there is endometriosis nodules or perforations and the treatment of other lesions such as bullous dystrophy [16]. For Riquet et al, surgery during menstrual period increases the sensitivity for the diagnosis of diaphragmatic lesions [17]. Many cases of recurrences are reported after talc pleurodesis or single pleural abrasion. For this reason, we must consider the etiopathogenic hypotheses including the existence of diaphragmatic defects and search for it [18]. Hormonal treatment blocks the hormonal contribution to the endometrial tissue and prevents further dissemination. When administered alone, it is associated with a significant rate of recurrence. Progestins and antigonadotropic (GnRH) were used. No product has shown its superiority, the choice depending primarily on the tolerance and the adverse effects [19].

## Conclusion

The occurrence of catamenial pneumothorax in woman should prompt a search for thoracic endometriosis. Our case draws its originality from the rare diaphragmatic localization of endometriosis.

## Competing interests

The authors declare no competing interests.
